# Supplementary material for: Examining the meaning and methodological characteristics of the systematized review label: A scoping review protocol
Source: PLoS One. 2023 Sep 8;18(9):e0291145. doi: 10.1371/journal.pone.0291145 (PMC10490901; doi:10.1371/journal.pone.0291145)
Supplement: S1 Appendix — (DOCX) [file pone.0291145.s002.docx]

### Appendix I: Data extraction instrument built in Google Sheets

| **Column heading** | **Input type** | **Options, if applicable** |
| --- | --- | --- |
| Citation | Open text | N/A |
| DOI | Open text | N/A |
| Publication year | Open text | N/A |
| Discipline | Dropdown | Health, Humanities, Social Sciences, Science, Multidisciplinary |
| Number of authors | Open text | N/A |
| Number of scholarly sources searched | Open text | N/A |
| Exact text of scholarly sources list | Open text | N/A |
| Grey literature searched | Dropdown | Yes, No, Unclear |
| Search date provided | Dropdown | Yes, No, Range provided |
| Citation searching | Dropdown | Yes, No |
| Reproducible search included | Dropdown | None, 1 DB, >1 DB |
| Protocol | Dropdown | Not mentioned, Mentioned, Published link provided |
| Date limit used | Dropdown | Yes, No |
| Range of limit used | Dropdown | 5 yrs or less, 6-10 years, 11-20 years, >20 years |
| Date limit justification reason | Open text | N/A |
| Other questionable limits found | Open text | N/A |
| Screening in duplicate | Dropdown | Yes, No, Unclear |
| Data extraction in duplicate | Dropdown | Yes, No, Unclear |
| Risk of Bias conducted | Dropdown | Yes, No, Unclear |
| Conducting guide | Dropdown | Guide or handbook referenced, published example referenced, None |
| Reporting guide | Dropdown | Guide or handbook referenced, published example referenced, None |
| Justification for selecting a systematized review mentioned | Dropdown | Yes, No |
| Exact text used in justification for choice of methodology | Open text | N/A |
| Citation provided for conceptualization of a systematized review (e.g. Grant & Booth, 2009) | Open text | N/A |
| Librarian involvement | Dropdown | Not mentioned, Librarian coauthor, Librarian mentioned in methods/acknowledgement |
| Additional comments | Open text | N/A |
